# Compensation of small data with large filters for accurate liver vessel segmentation from contrast-enhanced CT images

**DOI:** 10.1186/s12880-024-01309-1

**Published:** 2024-05-31

**Authors:** Wen Chen, Liang Zhao, Rongrong Bian, Qingzhou Li, Xueting Zhao, Ming Zhang

**Affiliations:** 1 Department of Medical Imaging, The First Affiliated Hospital of Xi’an Jiaotong University, Xi’an, China; 2 Taihe Hospital, Hubei University of Medicine, Shiyan, China

**Keywords:** Vessel segmentation, Image filtering, Markov random field

## Abstract

**Background:**

Segmenting liver vessels from contrast-enhanced computed tomography images is essential for diagnosing liver diseases, planning surgeries and delivering radiotherapy. Nevertheless, identifying vessels is a challenging task due to the tiny cross-sectional areas occupied by vessels, which has posed great challenges for vessel segmentation, such as limited features to be learned and difficult to construct high-quality as well as large-volume data.

**Methods:**

We present an approach that only requires a few labeled vessels but delivers significantly improved results. Our model starts with vessel enhancement by fading out liver intensity and generates candidate vessels by a classifier fed with a large number of image filters. Afterwards, the initial segmentation is refined using Markov random fields.

**Results:**

In experiments on the well-known dataset 3D-IRCADb, the averaged Dice coefficient is lifted to 0.63, and the mean sensitivity is increased to 0.71. These results are significantly better than those obtained from existing machine-learning approaches and comparable to those generated from deep-learning models.

**Conclusion:**

Sophisticated integration of a large number of filters is able to pinpoint effective features from liver images that are sufficient to distinguish vessels from other liver tissues under a scarcity of large-volume labeled data. The study can shed light on medical image segmentation, especially for those without sufficient data.

## Background

**Fig. 1:**
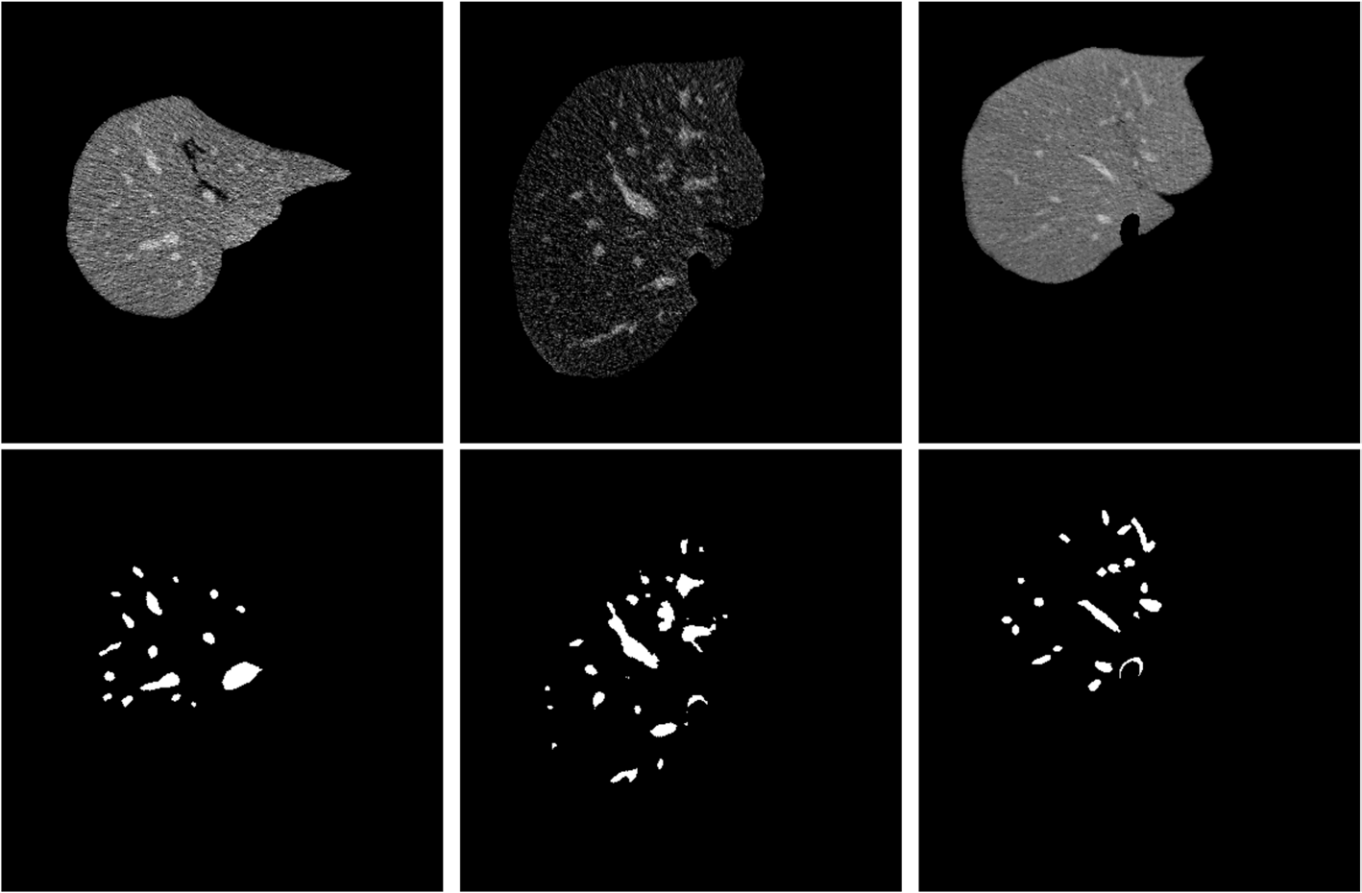
Vessel segmentation. The first row is the original images, while the second is the vessel masks obtained from 3D-IRCADb [22]

Liver vessel segmentation from computed tomography (CT) images is to pinpoint the pixels that comprise the vessels; see Fig. 1. Vessel segmentation is quite helpful in many clinical applications [1, 2], e.g., disease diagnosis, surgical planning, thermal ablation, etc. Hence, many computational approaches have been developed to solve this problem, both from the traditional machine learning perspective as well as the deep learning perspective, particularly the latter one.

The traditional machine-learning techniques that are borrowed for vessel segmentation include active contour or level set [3], graph cut [4, 5], extreme learning machine [6], vascular filters [7–9], and still many others [10–13]. These approaches can fish out vessels from CT images with moderate accuracy and time-saving. However, the segmentation can be easily leaked into the adjacent tissues. Besides, some of these approaches require careful initialization, parameter settings, or feature engineering. These limitations highly prevent the applicability of the aforementioned models.

Hence, deep learning-based approaches have been intensively explored and exploited to overcome these constraints because of their automatic feature learning characteristics. These approaches include convolutional neural network-based [14–16], recurrent neural network-based [17], a mixture of convolution and recurrent neural works [18], and integration of deep neural networks with conventional machine learning techniques [19, 20]. These deep learning-based models manifest remarkable improvement compared with the traditional approaches. However, they require large volumes of manually delineated images containing vessels. Unfortunately, delineating vessel masks with high fidelity is prohibitively difficult and time-consuming. The main obstacles preventing this goal are small size, irregular shape, low contrast and heavy noise; *cf.* Fig. 1. Hence, developing a model-driven but not data-starved approach is still very promising.

To this end, we develop a new computational model that borrows a large number of existing renowned image filters to distinguish vessels from other tissues and then use XGBoost [21] to classify each pixel as vessels or others. Finally, a refined Markov random field integrates neighborhood information to polish the results. Experimental results carried out on a widely used dataset 3D-IRCADb [22] show that our newly proposed model outperforms all existing traditional machine learning models, even better than deep learning-based models in most cases. Our model only requires a small number of labeled images to train the model but yields competitive or better results. The success reveals that many filters can compensate for the shortage of labeled data, which can be inspiring and promising for those tasks where high-quality data is challenging to obtain.

## Methods

**Fig. 2:**
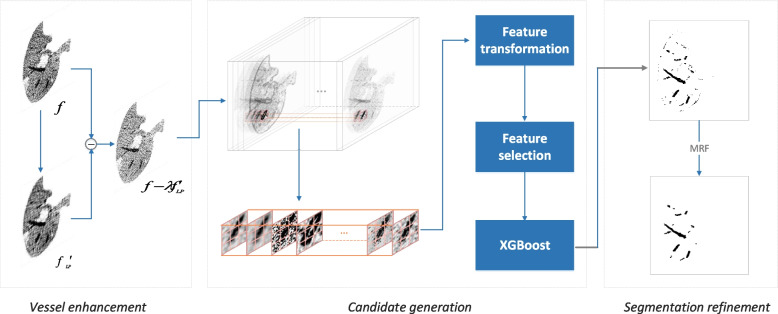
Diagram of the proposed liver vessel segmentation model. It composes vessel enhancement, candidate generation, and segmentation refinement. Vessel enhancement is achieved by fading out the background but strengthening boundary regions, candidate vessels are obtained by XGBoost feeding with features generated from extensive image filters, and refinement is fulfilled by a refined Markov random field

The proposed liver vessel segmentation model composes of three modules: vessel enhancement, candidate generation, and segmentation refinement; see Fig. 2. The details are as follows.

### Vessel enhancement

Two procedures are applied to the raw images to enhance the edges between vessel areas and other liver tissues, including calibration and contrast.

Calibration is necessary as the raw image may need to be clipped to the appropriate window for vessel analysis. To this end, we automatically determine the window center and width by a statistical approach. Precisely, the mean $\mu$ and standard deviation $\sigma$ of *vessel* intensities are determined. Then, the intensities of all images are clipped into an interval $[{\mu } - 3{\sigma }, {\mu } + 3 {\sigma }]$. These clipped intensities are further normalized to alleviate the systematic bias between different imaging devices by

$$\begin{aligned} f^{\prime }(x,y)=\alpha (f(x,y)-\mu )/\sigma )+c, \end{aligned}$$

where *f*(*x*, *y*) is the initial intensity of an image at position (*x*, *y*), $\alpha$ and *c* are used to transform the normalized values into gray scales from 0 to 255.

After calibration, the vessels are enhanced by

$$\begin{aligned} f^{\prime }(x,y)=f(x,y)-\lambda f(x,y)\circledast k(x,y), \end{aligned}$$

where *k*(*x*, *y*) is a kernel of a low-pass filter, $\lambda$ controls its magnitude, and $\circledast$ means convolution. This operation helps wash out many liver tissues and makes vessels stand out.

### Candidate generation

#### Feature transformation

The filters used to retrieve features from images include CLAHE (contrast limited adaptive histogram equalization) [23], Gabor filter [24], Gamma Correction [25], Gaussian filter [26], Hessian [7], Laplacian operator [27], Median filter [28], Mean filter [29], Minimum filter [30], Bilateral filter [31], Sobel operator [32], Canny edge detector [33], as well as the ten filters predefined in the imageFilter module of Pillow [34], which are BLUR, CONTOUR, DETAIL, EDGE_ENHANCE, EDGE_ENHANCE_MORE, EMBOSS, FIND_EDGES, SMOOTH, SMOOTH_MORE and SHARPEN. The mathematical definitions of these filters/operators are shown in Table 1.

**Table 1:** The filters and operators that are used to transform CT images

Operator	Definition
CLAHE [23]	$I^{k}=(k-1)CL(\sum p(k))$
Gabor [24]	$g(x,y)=\text {exp} \left( -\frac{x^{\prime 2}+\gamma ^{2}y^{\prime 2}}{2\sigma ^{2}}\right) \text {exp} \left( i \left( 2\pi \frac{x^{\prime }}{\lambda }+\psi \right) \right)$
Gamma [25]	$I^{\prime }(i,j)=\lambda I(i,j)^{\gamma }$
Gaussian [26]	$g(x,y)=\text {exp} \left( -\frac{x^2+y^2}{2\sigma ^2}\right)$
Hessian [7]	$\textbf{H}(I(i,j))=\textbf{J}(\nabla I(i,j))$
Laplacian [27]	$\Delta I(x,y)=\frac{\partial ^2I(x,y)}{\partial x^2}+\frac{\partial ^2I(x,y)}{\partial y^2}$
Median [28]	$I^{\prime }(i,j)=\text {med} \left( \textbf{K}_{(i,j)}(I(i,j); w,h \right)$
Mean [29]	$I^{\prime }(i,j)=\frac{1}{w*h}\sum \left( \textbf{K}_{(i,j)}(I(i,j); w,h \right)$
Minimum [30]	$I^{\prime }(i,j)=\text {min} \left( \textbf{K}_{(i,j)}(I(i,j); w,h \right)$
Bilateral [31]	$I^{\prime }(i,j)=\frac{1}{W_{p}}\sum _{i^{\prime },j^{\prime }\in \omega } I(i^{\prime },j^{\prime })f_{r}(\parallel I(i^{\prime },j^{\prime })-I(i,j)\parallel )g_s(\parallel (i^{\prime }-i)+(j^{\prime }-j)\parallel )$
Sobel [32]	$I^{\prime }=\sqrt{(\textbf{K}*I)^2+(\textbf{K}^{\text {T}}*I)^2}$
Canny [33]	TrackEdge(DoubleThreshold(GradientSuppression(Gradient(Smooth(*I*)))))
Pillow [34]	Predefined in the imageFilter module of the package

$CL(\cdot )$ is a contrast limited function, *g*(*x*, *y*) is the function to be convoluted to the image matrix *I* with *x* and *y* the distance between the current location and the interest point (*i*, *j*), $I^{\prime }(i,j)$ is the manipulated intensity of the original intensity *I*(*i*, *j*), $\textbf{K}$ is convolution kernel, $\textbf{H}(\cdot )$ is Hessian matrix, $\textbf{J}$ is Jacobian matrix, $x^{\prime }=x\text {cos}\theta +y\text {sin}\theta$, $y^{\prime }=y\text {cos}\theta -x\text {sin}\theta$, $W_p=\sum _{i^{\prime },j^{\prime }\in \omega } f_{r}(\parallel I(i^{\prime },j^{\prime })-I(i,j)\parallel )g_s(\parallel (i^{\prime }-i)+(j^{\prime }-j)\parallel )$, $f_r$ is an intensity smoothing function, $g_s$ is a coordinate smoothing function, $(f*g)$ means the convolution operation between *f* and *g*, *w* and *h* are the kernel width and height, $\gamma$, $\sigma$, $\lambda$ and $\psi$ are parameters

These filters have their unique merits in capturing features from images. Thus, the information obtained in this way is adequate to characterize vessels.

#### Context-aware vessel identification

Based on the filters, each pixel is represented by a *d*-dimensional vector containing its original intensity as well as all the values generated by the filters. Hence, the context as well as the vessel regions can be represented by a $n\times d$ vector with *n* the number of neighbors surrounding the interested pixel to be classified.

A pixel $F(i^{\prime },j^{\prime },k^{\prime })$ is deemed as an *h*-hop neighbor of the interest pixel *F*(*i*, *j*, *k*) if $\text {min}(|i-i^{\prime }|, |j-j^{\prime }|, |k-k^{\prime }|) \le h$, where *i*, *j* and *k* are the indices of a pixel, *i* and *j* are used to locate the pixel in a slice, and *k* is used to locate the slice in a volume. The *h* is set to 1, 2 and 3, resulting in the voxel size of $3\times 3\times 3$, $5\times 5\times 5$ and $7\times 7\times 7$, respectively. For the 2D situation, only *i* and *j* are considered.

The interested pixel as well as its neighbors form a voxel whose features are obtained from its constituent pixels, where its label is the mask of the central pixel. The features are obtained by using the above filters. The final features of the voxel are input into XGBoost [21] for feature selection and pixel classification.

### Segmentation refinement

The vessel segmentation is further refined by a Markov random field (MRF) [35] as the classification is only conducted on pixel level that ignores the correlation between pixels.

An MRF is a graph having ${G}=({V},{E})$, where *V* is the set of nodes (e.g., the pixels of an image), and *E* is the edges connecting the nodes in *V* (e.g., the adjacency pixels). For a random variable $v_{i}$ in *G*, the probability of $P(V=v_{i})$ is independent of other variables given its neighbors $N(v_{i})$ that is named as the Markov blanket. That being said,

$$\begin{aligned} P(V=v_{i}|V- v_{i})=P(V=v_{i}|N(v_{i}). \end{aligned}$$

Based on the Hammersley-Clifford theorem [36], it can be expressed as

$$\begin{aligned} P(V=v_{i}|N(v_{i}))=\frac{1}{Z}\text {exp}(-E(V=v_{i}|N(v_{i}))), \end{aligned}$$

where $E(\cdot )$ is an energy function and *Z* is the partition function computed by $Z=\sum _{v_{i}}E(v_{i})$. In this study $E(v_{i})$ is calculated by

$$\begin{aligned} E(v_{i})= & {} E_{\text {intensity}}(v_{i})+\lambda E_{\text {gradient}}(v_{i}) \\= & {} \sum \rho (u_{i}-v_{i},\sigma _{i}) + \lambda \sum \limits _{v_{j}\in N(v_{i})}\rho (v_{i}-v_{j},\sigma _{g}), \end{aligned}$$

where $u_{i}$ is the refined value of the variable $v_{i}$ and $\rho (x,\sigma )$ is the Lorentzian function [37] defined by

$$\begin{aligned} \rho (x,\sigma )=log \left( 1+\left( \frac{x}{\sigma }\right) ^{2}/2 \right) . \end{aligned}$$

By minimizing the energy function *E*, we obtained the refined segmentation of the vessels based on the pixel-wise classification results.

## Experiments

### Datasets

The well-known dataset 3D-IRCADb [22], scanned using contrast-enhanced computed tomography, is adopted for our model training and validation. In this dataset, all the masks of the liver, hepatic veins, portal veins, and arteries are available. Since 3D-IRCADb only contains 20 volumes (2,823 slices), it is suitable for traditional machine learning approaches but not deep learning-based models. It is because computational models should be trained in cases instead of slices so that training bias can be largely avoided. Thus, we will not make head-to-head comparisons with the deep learning models because of overfitting.

### Evaluation metrics

Four metrics are used to evaluate the performance, i.e., accuracy (Acc), sensitivity (Sen), Specificity (Spe), and dice similarity coefficient (DSC). They are defined as

$$\begin{aligned} Sen= & {} \frac{TP}{TP+FN}\\ Spe= & {} \frac{TN}{TN+FP}\\ Acc= & {} \frac{TP+TN}{TP+TN+FP+FN}\\ DSC= & {} \frac{2 \cdot TP}{FP+FN+2 \cdot TP} \end{aligned}$$

where true positives (*TP*) are vessel pixels classified correctly, false positives (*FP*) are pixels classified as vessels incorrectly, true negatives (*TN*) are pixels classified as non-vessels correctly, and false negatives (*FN*) are vessel pixels classified incorrectly. Among them, DSC is more meaningful as it is robust to imbalanced labels that are very common in vessel data.

### Performance qualification

#### Performance on 3D-IRCADb

**Fig. 3:**
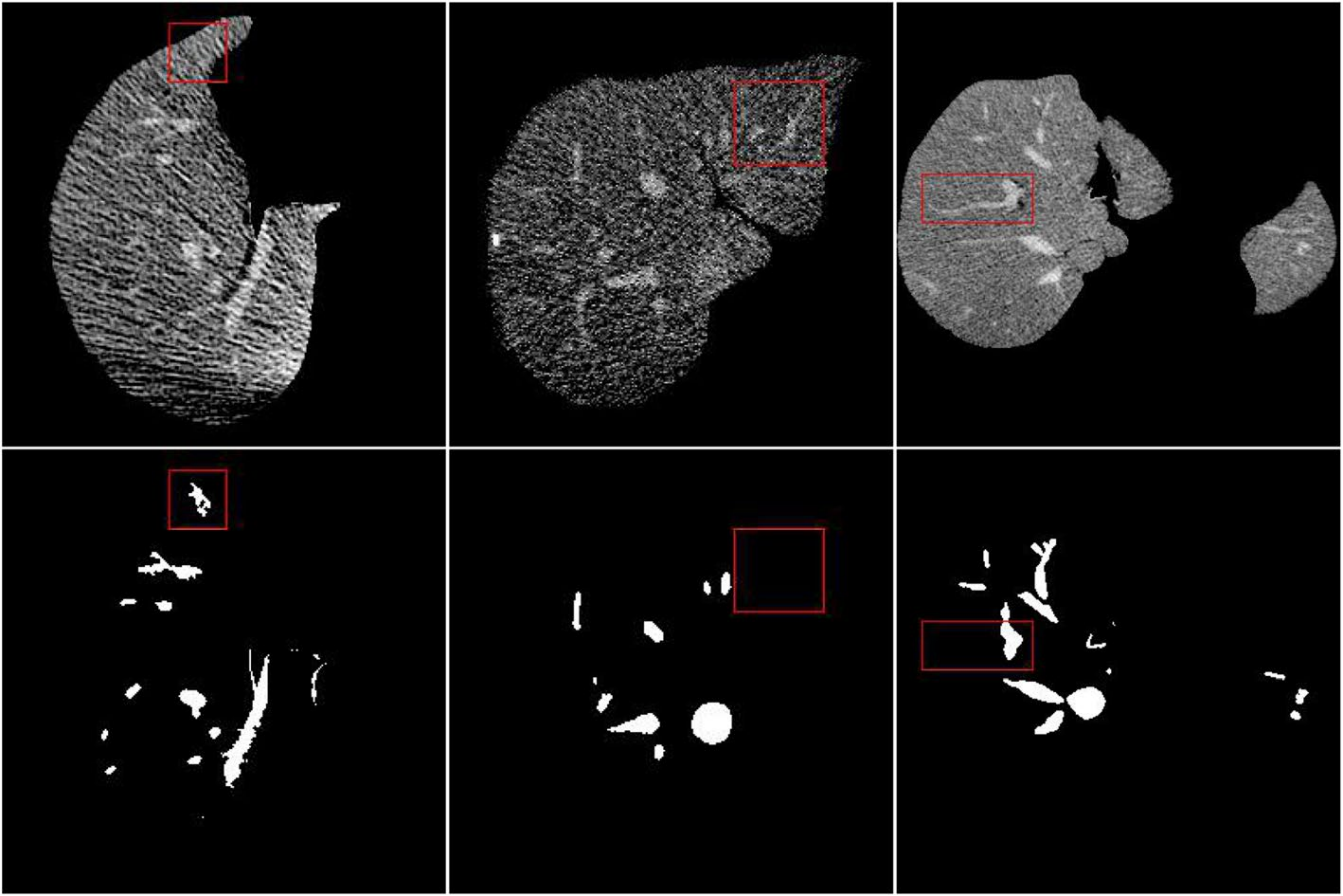
Examples of imperfect vessel labels. The red boxes highlight over-labeled, under-labeled, and wrongly-labeled masks

The performance of our model is evaluated through a rigorous five-fold cross-validation process. The dataset is partitioned into five folds at the scan level, with four folds (16 scans) designated for training and the remaining fold (4 scans) for testing. The training and testing are iterated across all folds to ensure comprehensive evaluation of all scans independently. On average, the DSC is 0.63 for all the volumes in 3D-IRCADb. However, this score is rarely reported by others. In addition, only partial volumes with top-performed results are reported by others as well. Therefore, we present the results obtained from 3D-IRCADb with the same number of volumes as others; c.f., Table 2. Results show that our model significantly outperforms existing approaches in terms of accuracy and specificity. Regarding sensitivity, our model is superior to others across all the cases, exhibiting an average lift of 2% when compared to the existing leading model. Notably, both sensitivity and DSC can be substantially influenced by the quality of reference masks and predictive accuracy. After carefully checking the labels of 3D-IRCADb, we have found a considerable portion of labels that are incorrectly masked. Taking Fig. 3, there have many over-labeled, under-labeled, and even wrongly-labeled masks. Since the number of vessel pixels is significantly smaller than that of non-vessel pixels, it is more sensitive to imperfect labels, thus the significant fluctuation of sensitivity.

**Table 2:** Performance comparison on 3D-IRCADb

Method	Dataset	Acc	Sen	Spe	DSC
Kitrungrotsakul et al. [16]	1 volume	-	0.90	-	0.92
Guo et al. [5]	8 volumes	0.97	0.66	0.98	-
Zhang et al. [13]	14 volumes	0.98	0.79	0.98	-
Lebre et al. [9]	20 volumes	0.97	0.69	0.98	-
U-Net [38]	20 volumes	0.99±1.1e-3	0.75±9.3e-2	0.99±2.2e-4	0.64±8.7e-2
TransUNet [39]	20 volumes	0.99±1.0e-3	0.73±7.5e-2	0.99±1.9e-4	0.62±7.6e-2
3D U-Net [40]	20 volumes	0.99±1.2e-3	0.67±1.4e-1	0.99±4.9e-4	0.60±6.2e-2
Ours	1 volume	0.99	0.94	0.99	0.93
	8 volumes	0.99±8.9e-4	0.85±1.1e-3	0.99±2.7e-4	0.76±9.3e-2
	14 volumes	0.99±9.7e-4	0.77±6.3e-2	0.99±2.1e-4	0.68±6.8e-2
	20 volumes	0.99±1.0e-3	0.71±8.7e-2	0.99±2.0e-4	0.63±5.9e-2

Note that, to ensure a fair comparison, we adhere to the standard settings for the number of testing volumes used in existing approaches: 1, 8, 14, and 20 volumes, respectively. The performance of each volume is evaluated using the five-fold cross-validation, and the results for *k* collective volumes are averaged on the *k* top-performed volumes.

#### Performance comparison with deep learning models

The proposed model is trained using multiple filters, necessitating only a small amount of labeled data intrinsically. Nonetheless, it may be less effective compared to deep learning-based models that are capable of automatic feature learning. To assess the efficacy of our proposed model, we evaluate its performance against state-of-the-art deep learning models, including U-Net [38], TransUNet [39], and 3D U-Net [40]. Detailed results presented in Table 2 reveal that our model is slightly inferior to U-Net but notably superior over TransUNet and 3D U-Net. We speculate that this discrepancy is primarily due to the increased parameters in the latter two models, particularly in the case of 3D U-Net.

#### Larger context improves segmentation

Different window sizes, i.e., 1, 3, 5 and 7, are used to capture the context information for vessel segmentation. To explore the impact of the context within a slice and between slices, we have considered the 2D and 3D scenarios. The performance of our model on 3D-IRCADb with various context window sizes are shown in Table 3. Clearly, a larger window of context consistently generates better segmentation results.

**Table 3:** Segmentation performance of our proposed model on 3D-IRCADb under various voxel size

Voxel	Acc	Sen	Spe	DSC
1$\times$1$\times$1	0.997	0.608	0.998	0.510
1$\times$3$\times$3	0.997	0.664	0.998	0.542
1$\times$5$\times$5	0.998	0.683	0.998	0.560
1$\times$7$\times$7	0.997	0.705	0.998	0.574
3$\times$3$\times$3	0.997	0.724	0.997	0.553
5$\times$5$\times$5	0.998	0.735	0.998	0.599
7$\times$7$\times$7	0.998	0.712	0.999	0.628
9$\times$9$\times$9	0.998	0.701	0.999	0.612

Results are obtained by five-fold cross-validation

**Fig. 4:**
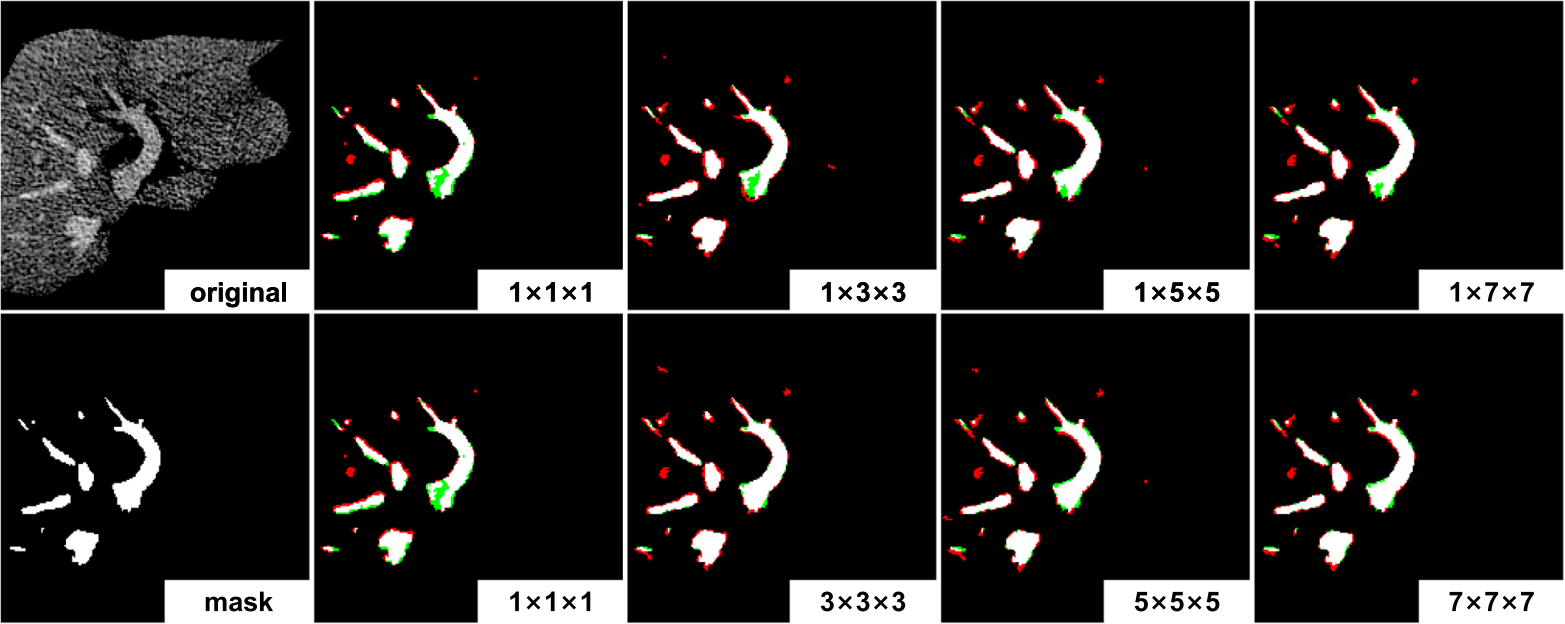
Vessel segmentation results obtained from various context ranges. The pixels in white are correctly predicted, the red are over predicted (i.e., false positive) and the green are under predicted (i.e., false negative). The mark “$i\times j\times k$” on a slice indicates the voxel size, where $i=1$ means the context in 2D, otherwise 3D

Figure 4 shows two examples of vessel segmentation with various window sizes. It can be observed that a larger window size generates complete internal regions and smoother edges of vessels. In contrast, small window size is prone to yield more isolated pixels or regions. Besides, the results obtained from 3D voxels are more tolerant to weakly connected regions between vessels than that generated from the 2D pixels.

It is essential to note that a larger voxel size does not always translate to better performance; see Table 3. This is due to the reduced influence of long-distance pixels on the central voxel of interest. Additionally, increasing voxel size substantially enlarges the feature dimension, potentially leading to issues such as the curse of dimensionality.

#### Markov random field refines segmentation

**Fig. 5:**
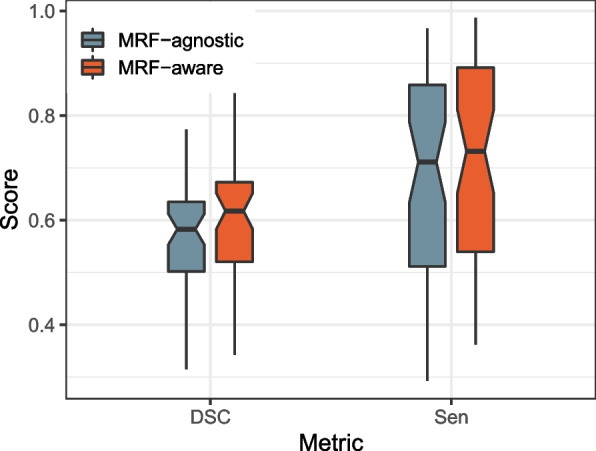
Performance comparison between MRF-aware and MRF-agnostic results. Note only the distribution of dice coefficient and sensitivity are shown here as others are very close to 1 that lose distinguishability

Although context information has been appended to the model of vessel segmentation, each pixel is predicted separately. Thus the connections of vessels in more extensive ranges are not captured. To this end, we borrow the MRF [35] model with a revised energy function to sharpen the distinction between vessels and non-vessels. The MRF-aware results improve the dice value by 3.1% on average for the 3D-IRCADb dataset (*p*-value $<2.2e-16$); see Fig. 5.

**Fig. 6:**
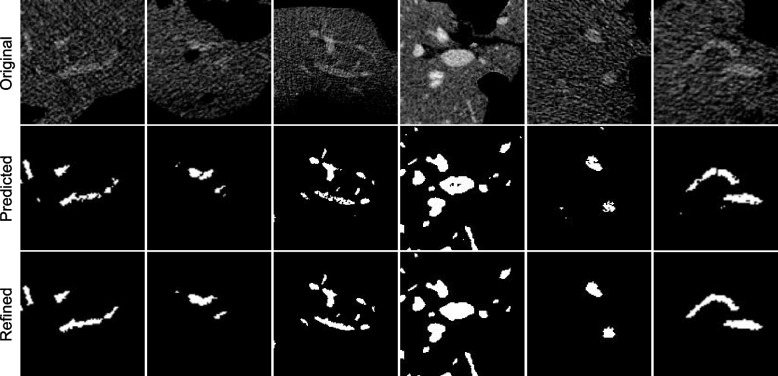
Examples of vessel segmentation improvements achieved by the MRF model. The first row contains the original images, the second is the results obtained without MRF refinement, and the third shows the purified results. It can be seen that MRF is able to remove isolates, fill holes, and bridge gaps

To demonstrate the improvements of the MRF model, we present six representative examples in Fig. 6. It is clear that the revised MRF model is able to remove isolated pixels or smaller regions, fill the holes in vessel regions, and bridge the gaps between separated vessel segments.

#### Association between critical filters and context

In this study, 22 filters are used to capture vessels’ information from various perspectives to compensate for the lack of data. However, not all filters are of equal importance to the model. To examine the association between the filters and the context size, we have retrieved the filters selected by XGBoost; see Table 4. Interestingly, only CLAHE, Gabor and Hessian are persistently important to the 2D-wise vessel segmentation. At the same time, most filters are kept for the 3D situation except a few presented in the Pillow package (the details are shown in Table 4). In addition, more filters are used in case the context range is more extensive. These observations consolidate our proposal of using multiple filters with broad context to segment vessels.

**Table 4:** Important filters to vessel segmentation with various context ranges

Filter	2D				3D														
	1	3	5	7	3			5					7						
					-1	0	1	-2	-1	0	1	2	-3	-2	-1	0	1	2	3
Origin						✓				✓			✓	✓	✓	✓	✓	✓	✓
CLAHE	✓	✓	✓	✓		✓		✓	✓	✓	✓	✓	✓	✓	✓	✓	✓	✓	✓
Gabor	✓	✓	✓	✓		✓			✓	✓	✓		✓	✓	✓	✓	✓	✓	✓
Gamma				✓						✓			✓		✓	✓	✓		✓
Gaussian				✓				✓	✓	✓	✓	✓	✓	✓	✓	✓	✓	✓	✓
Hessian	✓	✓	✓	✓		✓		✓	✓	✓	✓	✓	✓	✓	✓	✓	✓	✓	✓
Laplacian				✓						✓					✓	✓	✓		
Median				✓				✓	✓	✓	✓	✓	✓	✓	✓	✓	✓	✓	✓
Mean		✓		✓		✓		✓	✓	✓	✓	✓	✓	✓	✓	✓	✓	✓	✓
Minimum								✓	✓	✓	✓	✓	✓	✓	✓	✓	✓	✓	✓
Bilateral		✓	✓	✓				✓	✓	✓	✓	✓	✓		✓	✓	✓		✓
Sobel				✓						✓				✓	✓	✓	✓	✓	
Canny				✓						✓						✓			
PL_BLUR_^a^			✓	✓				✓	✓	✓	✓	✓	✓	✓	✓	✓	✓	✓	✓
PL_CONTOUR_										✓						✓			
PL_DETAIL_										✓			✓			✓			✓
PL_EDGE_ENHANCE_						✓				✓						✓			
PL_EDGE_ENHANCE_MORE_																✓			
PL_EMBOSS_																✓			
PL_FIND_EDGES_										✓									
PL_SHARPEN_										✓						✓			
PL_SMOOTH_				✓				✓	✓	✓	✓	✓	✓	✓	✓	✓	✓	✓	✓
PL_SMOOTH_MORE_			✓						✓	✓	✓		✓	✓	✓	✓	✓	✓	

^a^‘PL’ represents the Pillow package

The index ‘*i*’ ($i\in${-3, -2, -1, 0, 1, 2, 3}) indicates the position of a slice compared to the interest one (always marked as ‘0’) with negative the before and positive the behind. For the 2D situation, only one slice is presented, thus no such index is available

## Conclusion

Liver vessel segmentation is essential for clinical liver disease diagnosis and treatment. Hence great efforts have been made to solve this problem from the computational perspective. However, the performance of existing models is still far from satisfactory. The main reasons hindering vessel segmentation progress include small size, heavy noise, low contrast, and irregular shape. These difficulties further prevent the construction of large-volume and high-quality vessel segmentation data, making the computational models significantly under-fitted, particularly for deep learning models. To overcome the limitations, we propose a rich filter-based model to compensate for the scarcity of labeled data, of which the results are further refined by a Markov random field model. Experiments show that the proposed model significantly improves vessel segmentation without complicated models and extensive data. This study unveils that rich irrelevant filters are helpful for tasks having limited data, like vessel segmentation.

## Data Availability

The dataset supporting the conclusions of this article is available at https://www.ircad.fr/research/data-sets/liver-segmentation-3d-ircadb-01/, and the source codes can be found at https://github.com/lzhLab/veSeg/.
